# Supplementary material for: Epigenetic age provides insight into tissue origin in endometriosis
Source: Sci Rep. 2022 Dec 8;12:21281. doi: 10.1038/s41598-022-25416-7 (PMC9732286; doi:10.1038/s41598-022-25416-7)
Supplement: Supplementary file 1 — Supplementary Information. [file 41598_2022_25416_MOESM1_ESM.pdf]

Epigenetic age provides insight into tissue origin in endometriosis

Katie Leap, Iveta Yotova, Steve Horvath, and Julian A Martinez-Agosto

Supplemental Figure S1

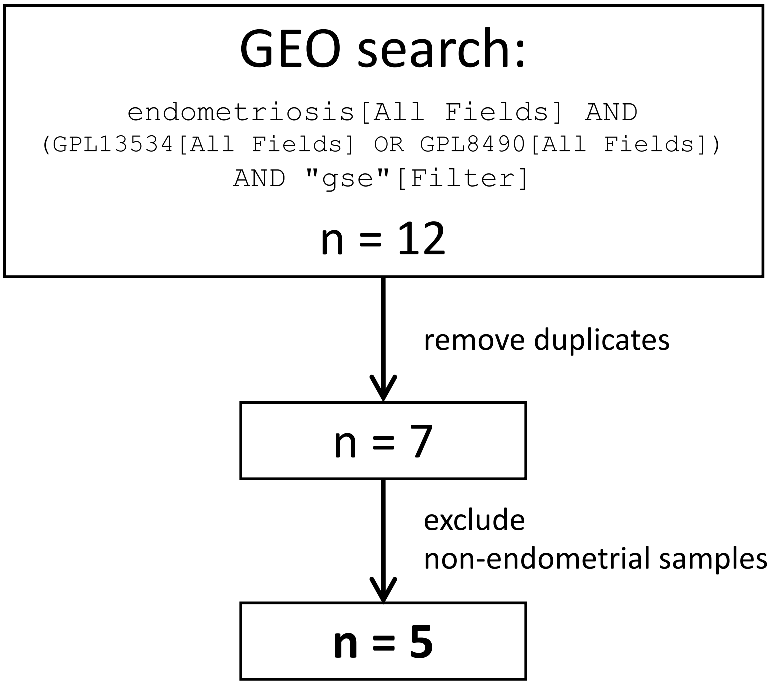

Supplemental Table S1

GEO Accession	Tissue Type	Sample Size	Platform	Analysis
GSE34355	medulloblastoma	15	GPL8490	
GSE73832	small intestine cancer	97	GPL13534	
GSE62231	paraganglioma	123	GPL8490	
GSE77269	hepatocellular carcinoma	57	GPL13534	metastatic cancer
GSE53051	multiple cancers	220	GPL13534	
GSE39279	non-small lung cancer	515	GPL13534	
GSE93589	endometrial cancer	9	GPL13534	
GSE81224	high grade serous ovarian cancer	20	GPL13534	
GSE43265	ovarian cancer	31	GPL8490	ovarian classifier
GSE26989	ovarian cancer	51	GPL8490	
GSE73948	endometrium (endometriosis)	24	GPL13534	
GSE73949	endometrium	17	GPL13534	
GSE90060	endometrium	34	GPL13534	endometriosis
GSE87621	stromal cells	9	GPL13534	
GSE47359	stromal cells	9	GPL8490	
GSE70783	intracranial germ cell tumors	77	GPL13534	teratomas

GPL8490: Illumina HumanMethylation27 BeadChip GPL13534: Illumina HumanMethylation450 BeadChip

Supplemental Figure S2

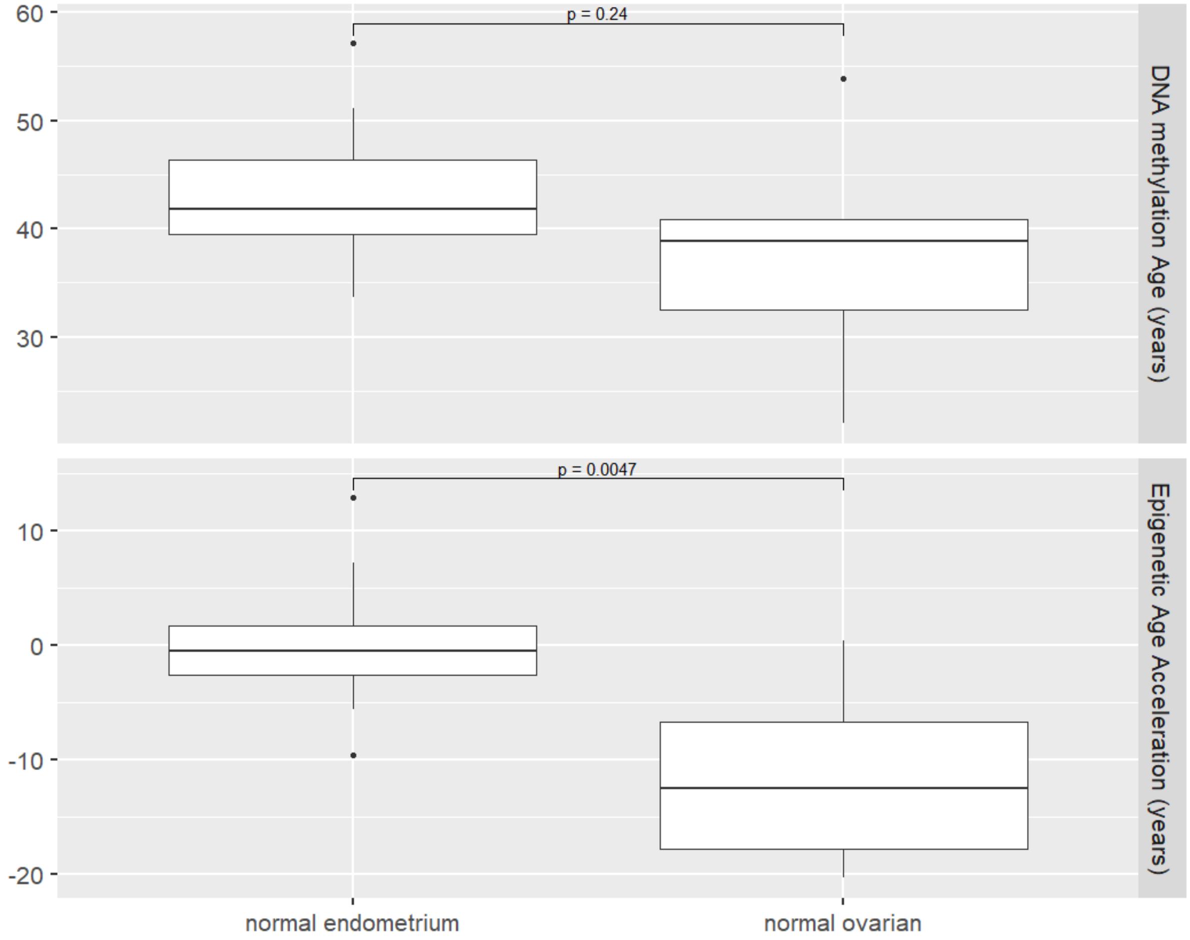

DNA methylation age is decelerated in normal ovarian tissue

Thirty-four (34) samples of non-pathological endometrium from GSE90060 and five (5) samples of non-pathological ovarian surface epithelial cells from GSE81224. P-values are calculated using the Wilcoxon rank-sum test.
